# The Efficacy and Safety of Dingkun Pill in Women with Polycystic Ovary Syndrome: A Systematic Review and Meta-Analysis of Randomized Controlled Trials

**DOI:** 10.1155/2022/8698755

**Published:** 2022-08-24

**Authors:** Bao Jin, Yang Zhang, Zongyu Zhang, Guorong Yang, Yujia Pan, Liangzhen Xie, Jiarui Liu, Wenjuan Shen

**Affiliations:** ^1^Department of Obstetrics and Gynecology, Heilongjiang University of Chinese Medicine, Harbin 150040, China; ^2^Department of Internal Medicine, First Affiliated Hospital, Heilongjiang University of Chinese Medicine, Harbin 150040, China; ^3^Department of Traditional Chinese Medicine, First Affiliated Hospital, Heilongjiang University of Chinese Medicine, Harbin 150040, China; ^4^Department of Biology, College of Life Science and Technology, Guangxi University, Nanning 530004, China; ^5^Department of Obstetrics and Gynecology, First Affiliated Hospital, Heilongjiang University of Chinese Medicine, Harbin 150040, China

## Abstract

**Objective:**

Dingkun Pill (DKP) is a proprietary Chinese medicine that has been utilized for patients with gynecological diseases, and its clinical application has been widely accepted in China. However, the effects of DKP on reproduction and metabolism in women with polycystic ovary syndrome (PCOS) have never been systematically evaluated. Our objective was to evaluate the efficacy and safety of DKP in treating reproductive and metabolic abnormalities with PCOS.

**Methods:**

We searched in PubMed, Embase, Web of Science, Cochrane Library, China National Knowledge Infrastructure, Wanfang Database, VIP Database, and Chinese Biomedical Literature Database up until January 2022 to identify randomized controlled trials (RCTs). The methodological quality of the included RCTs was estimated using the Cochrane collaboration risk-of-bias instrument, and the meta-analysis was performed using RevMan.

**Results:**

A total of 22 RCTs (including 1994 participants) were identified. DKP, combined with ovulation-inducing drugs (OID) or combined oral contraceptives (COC) was superior to OID or COC alone in improving the pregnancy rate (relative risk (RR) 1.84, 95% CI 1.62 to 2.11 and RR 1.38, 95% CI 1.16 to 1.64, respectively), ovulation rate (RR 1.38, 95% CI 1.03 to 1.84 and RR 1.23, 95% CI 1.11 to 1.37, respectively), endometrial thickness (weighted mean difference (WMD) 2.50, 95% CI 1.91 to 3.09 and WMD 0.62, 95% CI 0.08 to 1.16, respectively), luteinizing hormone (WMD −1.93, 95% CI −2.80 to−.07 and WMD −1.79, 95% CI −2.66 to−0.92, respectively), and testosterone (standardized mean difference (SMD) −2.12, 95% CI −3.01 to−1.24 and SMD −1.21, 95% CI −1.64 to−0.78, respectively). DKP combined with COC led to a greater improvement in homeostasis model assessment-*β* (WMD 20.42, 95% CI 16.85 to 23.98) when compared with COC alone. There was a significant difference between DKP and COC in terms of decreasing total cholesterol (WMD −0.37, 95% CI −0.72 to−0.02), triacylglycerol (WMD −0.85, 95% CI −1.50 to−0.20), and free fatty acid (WMD −130.00, 95% CI −217.56 to−42.22). However, DKP did not affect the follicle stimulating hormone, fasting blood glucose, fasting insulin, body mass index, waist-to-hip ratio, high-density lipoprotein cholesterol, or low-density lipoprotein cholesterol. Adverse reactions were more common in COC alone compared to DKP and COC in combination (RR 0.22, 95% CI 0.07 to 0.63).

**Conclusion:**

DKP shows promise in modifying reproductive and metabolic parameters in patients with PCOS and may be used as a primary choice in conventional or complementary therapies for PCOS. The quality of the evidence analyzed was suboptimal, and therefore, our results should be interpreted cautiously. More prospective large-scale and well-designed RCTs, as well as longer intervention durations are required in the future to draw more reliable conclusions.

## 1. Introduction

Polycystic ovary syndrome (PCOS) is a common gynecologic endocrine disorder that is generally considered to be the leading cause of anovulatory infertility [1], and it affects 6% to 20% of reproductive-age women [2]. PCOS is characterized by menstrual dysfunction, hypo-ovulation/anovulation, hyperandrogenism, and polycystic ovaries [3]. In addition to reproductive disruption, women with PCOS are prone to metabolic disorders, including insulin resistance (IR), impaired glucose tolerance, and dyslipidemia, and they are at an increased risk of developing type 2 diabetes mellitus [4]. In the U.S. alone, the cost of diagnosing, treating, and caring for patients with PCOS was estimated to be $8 billion yearly in 2020, which places an immense financial burden on both the patient's family and society as a whole [5]. Therefore, the effective management and treatment of PCOS can contribute to improving public health.

Western medicine management for PCOS includes ovulation-inducing drugs (OID), insulin sensitizers, combined oral contraceptives (COC), antiandrogens, and/or antiobesity medications aiming at restoring menstruation and improving pregnancy, decreasing androgen levels, lowering IR, and reducing weight [6]. However, they have some potential side effects. Although clomiphene citrate achieves an ovulation rate up to 70% to 80%, the clinical pregnancy rate is only 30% to 40%, and patients are at risk of multiple pregnancies [7]. Letrozole has a short half-life (42 h) and is quickly excreted from the body, thus resulting in the inability to form a dominant follicle [8]. Patients taking Diane-35 or metformin may suffer from abnormal uterine bleeding, gastrointestinal disturbances, and other adverse reactions [9]. Thus, an increasing number of PCOS patients have turned to complementary and alternative therapy to improve their health. According to a recent survey, 70.4% of obstetricians and gynecologists/reproductive doctors in China use traditional Chinese medicine in the treatment of PCOS [10].

Dingkun Pill (DKP) is a traditional Chinese patent herbal medicine originating from the *Golden Mirror of Medicine* written by Wu Qian in the Qing Dynasty and is officially listed in the Chinese Pharmacopoeia [11]. It is composed of 30 Chinese herbals and animal products, including red ginseng (*Radix Ginseng Rubra*), pilose antler (*Cornu Cervi Pantotrichum*), saffron (*Stigma Croci*), debarked peony root (*Radix Paeoniae Alba*), Chinese angelica (*Radix Angelicae Sinensis*), prepared rehmannia root (*Radix Rehmanniae Preparata*), ass hide glue (*Colla Corii Asini*), etc. For centuries, DKP has been used in traditional Chinese medicine to treat gynecological diseases because the combination of these ingredients is thought to nourish the liver and kidney, regulate menstruation, relieve Qi stagnation, benefit Qi, and nourish the blood. Among the traditional Chinese patent herbal medicine used for PCOS, DKP ranks first [10], and an increasing number of animal experiments and clinical studies have demonstrated the reliable efficacy of DKP [12–15]. In experimental studies, DKP and its main active ingredients were found to regulate the reproductive hormone levels in rats with PCOS, decrease the expression of vascular endothelial growth factor in the ovary, and increase the expression of homeobox gene A10 (HOXA10) in the uterus, thereby facilitating uterine receptivity [16, 17]. According to Gao's study, the mechanism of DKP in the treatment of PCOS might be associated with multiple signaling pathways, such as the PI3K-Akt signaling pathway, serotonergic synapses, steroid hormone biosynthesis, and ovarian steroidogenesis, suggesting that DKP can treat PCOS through multiple targets [18]. Regarding the effect of DKP in PCOS, the available clinical data suggest that DKP plays a role in regulating the menstrual cycle, promoting ovulation, increasing the pregnancy rate, and enhancing the function of the hypothalamus-pituitary-ovary axis (HPOA) [19, 20]. Moreover, DKP has also been used in PCOS patients with IR and lipid metabolism abnormalities [21, 22]. As a traditional Chinese patent herbal medicine, DKP has the advantages of easy access, convenient administration, and wide acceptance. Hence, it has great potential for popularization. However, as far as we know, there has been no systematic evaluation of the efficacy and safety of DKP in the treatment of reproductive and metabolic abnormalities in women with PCOS and whether this medicine represents an ideal form of complementary and alternative therapy. Thus, we conducted a systematic review and meta-analysis of available RCTs to provide a reliable basis for the treatment of PCOS.

## 2. Materials and Methods

This systematic review was conducted and reported according to the preferred reporting items for systematic reviews and meta-analysis (PRISMA) statement guidelines [23] and was registered in PROSPERO (CRD42022298220).

### 2.1. Search Strategy

The systematic literature search was performed in the following databases: PubMed, Embase, Cochrane Library, Web of Science, China National Knowledge Infrastructure (CNKI), Wanfang Database, VIP Database, and the Chinese Biomedical Literature Database (CBM) from their inception to 1 January, 2022. Key words in the literature retrieval included “Dingkun pill,” “Dingkun Dan,” “polycystic ovary syndrome,” “polycystic ovarian syndrome,” “PCOS,” and related synonyms (the full details of the search strategy are given in Table S1 in the Supplementary Materials). No limits were applied to language or publication status. The references of significant studies were searched manually for possible relevant literature, and conference compilations supplemented the electronic searches.

### 2.2. Eligibility Criteria

The inclusion criteria was as follows: (a) subjects diagnosed with PCOS regardless of race and age, (b) the intervention group was treated with DKP or DKP combined with the control group's intervention. The control group was treated with Western medicine, placebo, or blank and with an unlimited dose and course of treatment. (c) The study was an RCT. The exclusion criterion was the literature in which relevant data could not be obtained and data were still not available after contacting the authors.

### 2.3. Outcome Measures

As improving reproduction is the core in treating PCOS, the primary outcome measure was defined as reproductive indexes, including pregnancy rate, ovulation rate, and endometrial thickness. The secondary outcome measures were defined as hormone parameters—including luteinizing hormone (LH), follicle stimulating hormone (FSH), and testosterone (T)—metabolic indexes—including fasting blood glucose (FBG), fasting insulin (FINS), and homeostasis model assessment-*β* (HOMA-*β*)—lipid profiles—including total cholesterol (TC), triacylglycerol (TG), high-density lipoprotein cholesterol (HDL-C), low-density lipoprotein cholesterol (LDL-C), and free fatty acid (FFA)—and anthropometric indices—including body mass index (BMI) and waist-to-hip ratio (WHR). Adverse reactions were also included as outcomes.

### 2.4. Literature Screening and Data Extraction

Based on the search strategy presented above, the titles and abstracts of the identified articles were read for preliminary screening after eliminating duplicates. The full texts were then read during rescreening in accordance with the inclusion and exclusion criteria established previously to identify the included articles. Data extraction was performed independently by two reviewers, and disagreement was resolved by discussion. The following information was extracted from the included RCTs: (1) the characteristics of the articles, including primary author, publication year, language, and study design, (2) participants' characteristics, including mean age, sample size, and criteria used to define PCOS, (3) the details of interventions and comparison methods, including the type and treatment duration, (4) every outcome measure, and (5) adverse reactions. To ensure that the data were complete and accurate, we contacted the authors via telephone or e-mail regarding missing data.

### 2.5. Quality Assessment

Two reviewers independently assessed the methodological quality of eligible RCTs using the Cochrane collaboration risk-of-bias instrument [24]. Factors were related to bias risk-included random sequence generation, allocation concealment, the blinding of participants and personnel, the blinding of outcome assessment, incomplete outcome data, selective reporting, and other biases. There were three levels used to assess the methodological quality: the low risk of bias, the high risk of bias, and an unclear risk of bias. Then, we used the grading of recommendations, assessments, development, and evaluation (GRADE) [25] system (pro 3.6.1) to evaluate the quality of evidence derived from our systematic review for primary outcomes separately, including the risk of bias, indirectness, inconsistency, imprecision, and publication bias. All discrepancies and disagreements were resolved by consensus or by discussion with the corresponding author.

### 2.6. Statistical Analysis

All data syntheses were performed using the Review Manager software version 5.3. Dichotomous data were presented as the risk ratio (RR) and continuous data as the weighted mean difference (WMD) or standardized mean difference (SMD), both with a 95% confidence interval (CI). Heterogeneity across the studies was tested by the Cochrane Q-test and I^2^ statistic. If I^2^ ≤ 50% and *P* ≥ 0.10, a fixed effects model was used. Otherwise, a random effects model was used. To determine the stability of the meta-analysis results, a sensitivity analysis was conducted to explore heterogeneity because of extreme data. A funnel plot was used to assess publication biases.

## 3. Results

### 3.1. Study Selection

**Figure 1:**
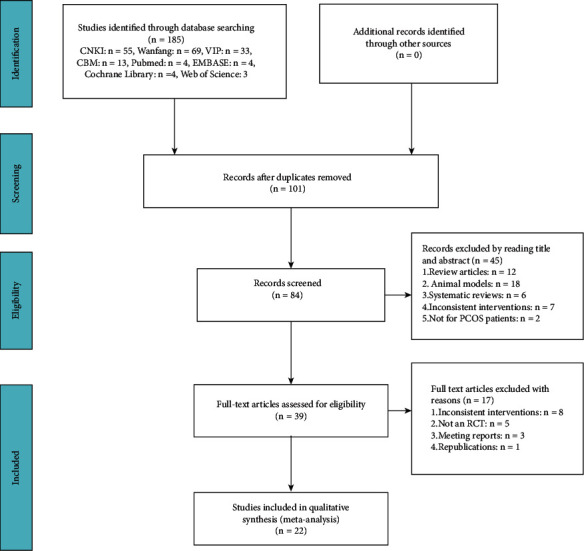
Flow diagram of the study selection process.

Originally, 185 articles were identified in the database through the search strategy, and it was reduced to 84 records after duplicates were removed. After reviewing the titles, abstracts, and full-text articles, a total of 22 RCTs in 24 publications [10, 19, 21, 22, 26–45], including 1994 women with PCOS, satisfied our inclusion criteria (Figure 1).

### 3.2. Characteristics of the Included Studies

The summarized characteristics of the 22 RCTs and the 1994 participants are shown in Table 1. All of the RCTs were conducted in China, with the sample size ranging from 60 to 210 participants, and most of them were 20 to 39 years old. Studies were published between 2012 and 2021. Most of the included RCTs used the Rotterdam criteria [46] to define PCOS, while three RCTs [36, 39, 41] used Obstetrics and Gynecology, Chinese Obstetrics and Gynecology Association, and Guidelines for the diagnosis and treatment of PCOS in China, respectively, and six RCTs [29, 31, 34, 35, 37, 40] only reported the diagnosis of PCOS and did not clearly describe the diagnostic criteria. The interventions were DKP alone or in combination with OID or COC, and the controls were OID or COC. Twenty of the 22 RCTs [19, 25–43] were 2-arm studies, and the remaining two RCTs were 3-arm studies. Data from the 3-arm study, divided into DKP vs. COC and DKP + COC vs. COC, were included in the meta-analysis. The duration of treatment was one month in two RCTs [33, 39], three months in 11 RCTs [10, 19, 21, 22, 28–30, 32, 40–44], six months in two RCTs [26, 38], and until pregnancy in the rest. Pregnancy rate and endometrial thickness were the most common outcomes followed by hormone parameters. Ten RCTs [10, 19, 21, 22, 26, 28, 33, 38, 39, 42–44] reported on adverse reactions.

### 3.3. Risk of Bias of Individual Studies

**Figure 2:**
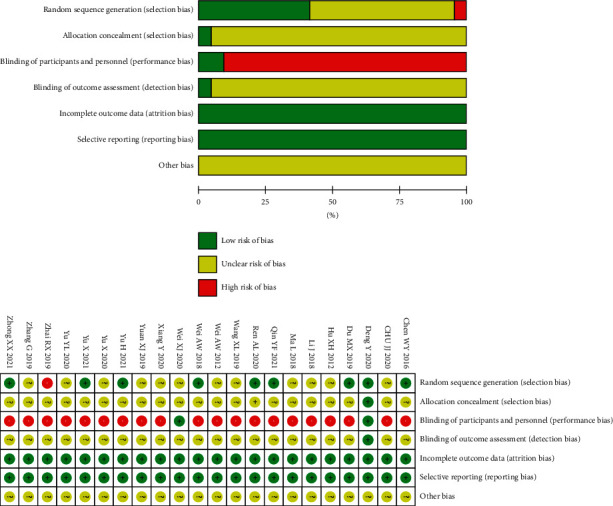
The risk of bias for the included studies shown as low risk of bias (+), high risk of bias (−), and unclear risk of bias (?).

Figure 2 summarizes the risk of bias of the included trials based on different quality domains using the Cochrane collaboration instrument. Nine trials [10, 21, 22, 25, 29, 30, 33, 37, 41–43] reported random sequence generation using a random number table or software and thus had a low risk, 12 trials [19, 26–28, 31, 32, 34–36, 38–40] only mentioned “random” but were missing details regarding the randomization methods and thus had an unclear risk, and one trial [38] had a high risk. Only one trial [10, 21, 22] had a low risk when considering allocation concealment. In terms of the blinding of participants or personnel, two trials [10, 19, 21, 22] had a low risk. The outcome assessors were blind in one trial [10, 21, 22] using triple-blinding, and the other 21 trials had an unclear risk. There was no risk of bias for incomplete outcome data or for selective reporting. All trials appeared to have an unclear risk for other biases.

### 3.4. Findings from the Meta-Analysis

#### 3.4.1. Effects on Reproductive Indexes

**Figure 3:**
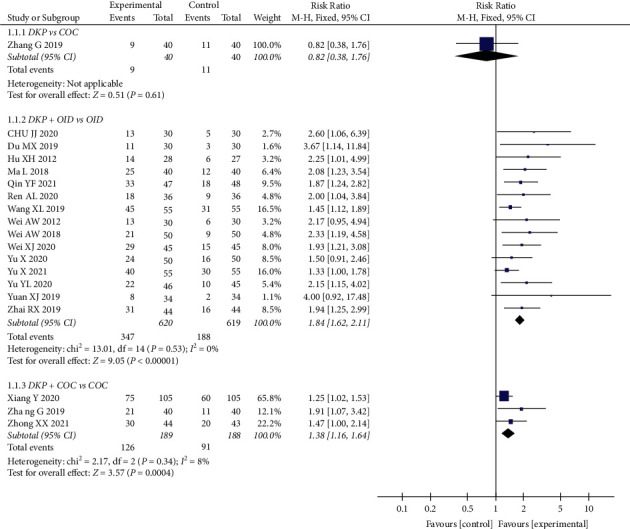
Meta-analyses of the effects of DKP on the pregnancy rate.

The effects of DKP on pregnancy rate was assessed in 18 RCTs (1616 participants). The pooled results showed that the combination of DKP + OID was superior to OID alone in increasing the pregnancy rate (RR: 1.84, 95% CI: 1.62 to 2.11, *P* < 0.00001). Compared with the COC groups, there was a significant improvement in pregnancy rate in the DKP + COC groups (RR: 1.38, 95% CI: 1.16 to 1.64, *P*=0.0004). However, a comparison of DKP with COC did not show a significant difference in pregnancy rate (RR: 0.82, 95% CI: 0.38 to 1.76, *P*=0.61) (Figure 3).

**Figure 4:**
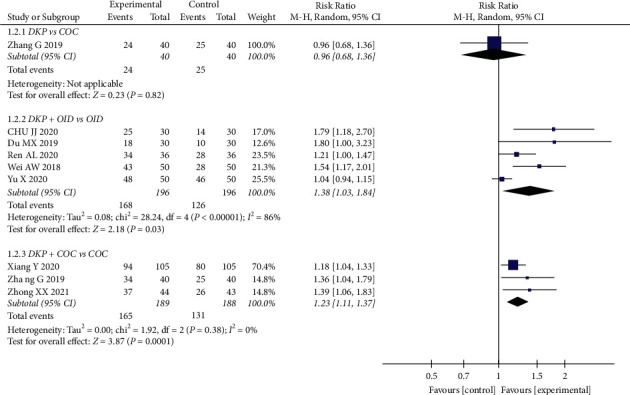
Meta-analyses of the effects of DKP on the ovulation rate.

In terms of ameliorating the ovulation rate, eight RCTs, including 769 participants, indicated that DKP plus OID or COC for PCOS was better than using OID or COC alone (OID: RR: 1.38, 95% CI: 1.03 to 1.84, *P*=0.03; COC: RR: 1.23, 95% CI: 1.11 to 1.37, *P*=0.0001). There was no clear difference between DKP and COC in terms of ovulation rate (RR: 0.96, 95% CI: 0.68 to 1.36, *P*=0.82) (Figure 4).

**Figure 5:**
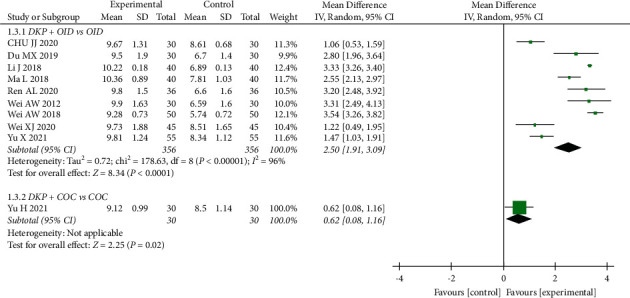
Meta-analyses of the effects of DKP on the endometrial thickness.

There were ten RCTs (772 participants) assessing the effects of DKP on endometrial thickness. Compared with OID alone, the combination DKP + OID significantly improved the endometrial thickness of PCOS patients (WMD: 2.50, 95% CI: 1.91 to 3.09, *P* < 0.00001). Furthermore, the combination of DKP + COC also significantly increased endometrial thickness (WMD: 0.62, 95% CI: 0.08 to 1.16, *P*=0.02) (Figure 5).

#### 3.4.2. Effects on Hormone Parameters

**Figure 6:**
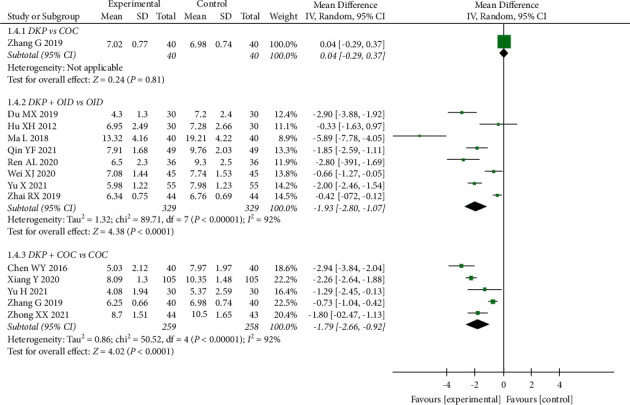
Meta-analyses of the effects of DKP on LH.

Thirteen trials totaling 1175 women were used in a meta-analysis of the effects of DKP on LH level. Compared with OID alone, DKP + OID significantly decreased LH (WMD: −1.93, 95% CI: −2.80 to−1.07, *P* < 0.0001). Similarly, the combination of DKP and COC was superior in reducing LH compared to COC alone (WMD: −1.79, 95% CI: −2.66 to−0.92, *P* < 0.0001). There was no significant difference between DKP versus COC in reducing LH (WMD: 0.04, 95% CI: –0.29 to−0.37, *P*=0.81) (Figure 6).

**Figure 7:**
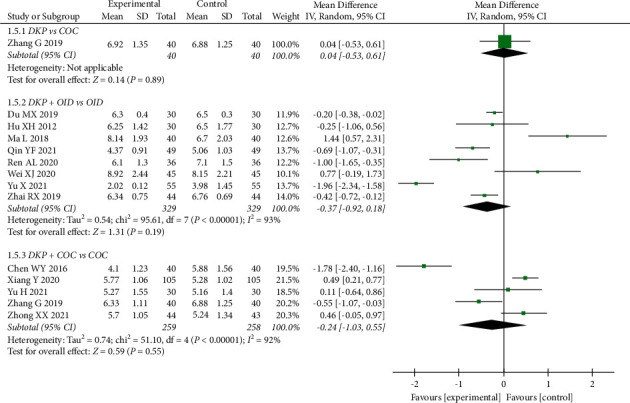
Meta-analyses of the effects of DKP on FSH.

Figure 7 shows the meta-analysis of FSH in 13 RCTs with a total of 1175 patients. DKP did not appear to have a significant effect on improving FSH because no statistically significant differences were seen: DKP + OID versus OID (WMD: −0.37, 95% CI: −0.92 to 0.18, *P*=0.19), DKP + COC versus COC (WMD: −0.24, 95% CI: −1.03 to 0.55, *P*=0.55), and DKP versus COC (WMD: 0.04, 95% CI: −0.53 to 0.61, *P*=0.89). However, a significant difference in the level of FSH was found in the subgroup analysis stratified by the duration of the intervention. In the stratified analysis, interventions lasting three months or more had a significant effect on FSH levels (WMD: −0.40, 95% CI: −0.68 to−0.12, *P*=0.006). The details of the subgroup analyses are shown in the Supplementary Materials.

**Figure 8:**
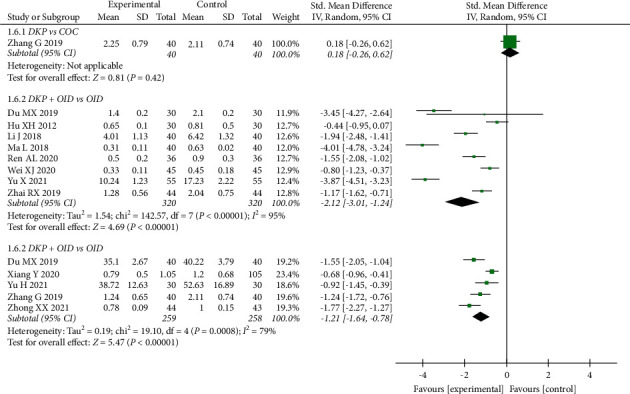
Meta-analyses of the effects of DKP on T.

A meta-analysis of 13 trials (1157 patients) found that T decreased more after DKP plus OID or COC treatment in comparison with OID or COC alone (OID: SMD: −2.12, 95% CI: −3.01 to−1.24, *P* < 0.00001; COC: SMD: −1.21, 95% CI: −1.64 to−0.78, *P* < 0.00001). On the contrary, the comparison of the DKP groups with the COC groups did not show a significant difference in T (SMD: 0.18, 95% CI: −0.26 to 0.62, *P*=0.42) (Figure 8).

#### 3.4.3. Effects on Metabolic Indexes

**Figure 9:**
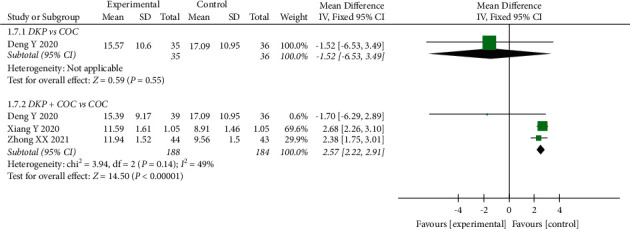
Meta-analyses of the effects of DKP on FINS.

The effects of DKP on FINS were assessed in 3 RCTs (372 patients). There was a significant decline in the FINS level after COC intake in comparison with DKP + COC treatment (WMD: 2.57, 95% CI: 2.22 to 2.91, *P* < 0.00001), while no significant difference was found between DKP and COC on FINS (WMD: −1.52, 95% CI: −6.53 to 3.49, *P*=0.55) (Figure 9).

One RCT reported FBG as an outcome measure and did not observe any significant change in FBG in PCOS patients after DKP treatment compared to COC treatment (WMD: 0.10, 95% CI: −0.09 to 0.29, *P*=0.31). In addition, compared with the COC group, there were no significant differences in FBG in the DKP + COC group (WMD: 0.10, 95% CI: −0.15 to 0.35, *P*=0.43) (Table 2).

**Figure 10:**
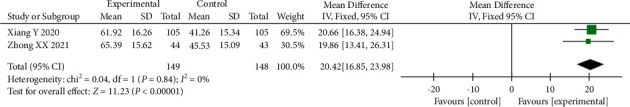
Meta-analyses of the effects of DKP on HOMA-*β*.

When we combined data from two studies (297 patients), a significant increase in HOMA-*β* was observed in DKP + COC treatment (WMD: 20.42, 95% CI: 16.85 to 23.98, *P* < 0.00001) (Figure 10).

#### 3.4.4. Effects on Lipid Profiles

As illustrated in Table 2, DKP in PCOS patients showed a significant reduction in serum TC (WMD: −0.37, 95% CI: −0.72 to−0.02, *P*=0.04), TG (WMD: –0.85, 95% CI: −1.50 to−0.20, *P*=0.01), and FFA (WMD: −130.00, 95% CI: −217.56 to−42.44, *P*=0.004) levels versus the COC group. However, COC improved the serum concentration of HDL-C compared to the DKP group (WMD: −0.35, 95% CI: −0.55 to−0.15, *P*=0.0008). There was no significant change in TC (WMD: 0.18, 95% CI:−0.16 to 0.52, *P*=0.31), TG (WMD: −0.19, 95% CI: −1.02 to 0.64, *P*=0.65), LDL-C (WMD: 0.21, 95% CI: −0.11 to 0.53, *P*=0.20), HDL-C (WMD: −0.04, 95% CI: −0.29 to 0.21, *P*=0.75), or FFA (WMD: −67.00, 95% CI: −157.12 to 23.12, *P*=0.15) when DKP + COC was administered (Table 2).

#### 3.4.5. Effects on Anthropometric Indices

BMI and WHR were measured in only one trial, and there were no significant changes in BMI or WHR for DKP alone (BMI: WMD: 0.70, 95% CI: −1.81 to 3.21, *P*=0.59; WHR: WMD: 0.00, 95% CI: −0.03 to 0.03, *P*=1.00) or for DKP + COC (BMI: WMD: 0.60, 95% CI: −1.92 to 3.12, *P*=0.64; WHR: WMD: −0.01, 95% CI: –0.04 to 0.02, *P*=0.47) (Table 2).

### 3.5. Adverse Reactions

**Figure 11:**
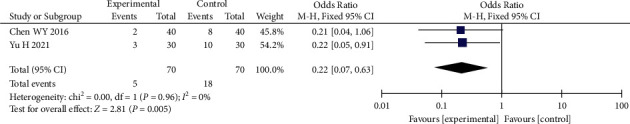
Forest plot for overall adverse reactions.

Ten trials [10, 19, 21, 22, 27, 29, 34, 39, 40, 43–45] recorded adverse events, of which eight trials reported that there were no adverse reactions. In the other two studies [43, 44], statistical analysis showed that DKP + COC was associated with fewer adverse events compared to COC alone (RR = 0.22, 95% CI: 0.07 to 0.63, *P*=0.005) (Figure 11). The results of Chen et al. [43] showed that adverse reactions occurred in the intervention group (mild nausea in 1 case and mild breast pain in 1 case) and the control group (mild nausea in 3 cases, mild breast pain in 2 cases, interphase hemorrhage in 1 case, and mild headache in 2 cases). In Yu's study [44], three patients had adverse reactions in the DKP + COC group, including TC elevation (*n* = 2) and direct bilirubin elevation (*n* = 1), and ten patients had adverse reactions in the COC group, including direct bilirubin elevation (*n* = 3), glutamic pyruvic transaminase elevation (*n* = 1), apolipoprotein A1 elevation (*n* = 1), TC elevation (*n* = 2), TG elevation (*n* = 2), and breast pain (*n* = 1). All the above adverse reactions were mild, and no serious adverse events were observed in the included trials.

### 3.6. Sensitivity Analysis

When the heterogeneity was high, we performed a sensitivity analysis. The results indicated that there was no significant change in the effect size of endometrial thickness, LH, or T after the one-by-one exclusion of the included literature, which confirmed the stability and reliability of the meta-analysis. Furthermore, removing Xiang et al. [41], whio investigated the influence of DKP on T, led to a decrease in heterogeneity, while the result remained significant (SMD: −1.37, 95% CI: −1.73 to−1.02, *P*=0.00001, I^2^ = 49%). However, the result of FSH was not robust, and removing Ma et al. [29] resulted in a positive overall effect (WMD: −0.59, 95% CI: −1.13 to−0.05, *P*=0.003).

### 3.7. Publication Bias

**Figure 12:**
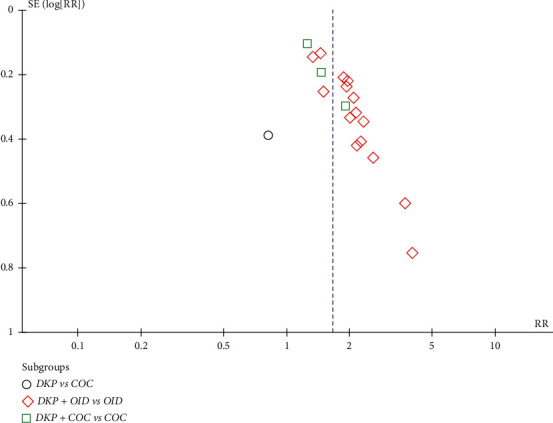
The funnel plot of the literature.

A funnel plot was used to evaluate publication bias, which showed that the symmetry between different studies was poor and that publication bias existed (Figure 12).

### 3.8. Quality of the Evidence

Table 3 shows a summary of the quality of evidence grades for selected primary outcomes. The quality of the evidence was downgraded to low or very low certainty according to the GRADE system. The main reason for this degradation was the limitations of the original studies because of the lack of randomization allocation and blinding, the unexplained heterogeneity between studies in the estimates of the treatment effects, the number of patients included being less than 400, and the publication bias.

## 4. Discussion

### 4.1. Main Results

The present study is the latest and most comprehensive systematic review of the effects of DKP on reproduction and metabolism in PCOS patients, including 22 RCTs (1994 participants). In this review, DKP was shown to significantly ameliorate 1) reproduction issues as evidenced by increased pregnancy rate, ovulation rate, and endometrial thickness, 2) hormone imbalances as assessed by decreased LH and T, 3) metabolic disorders as assessed by increased HOMA-*β*, and 4) lipid profile changes as evidenced by decreased TC, TG, and FFA in PCOS patients. However, DKP combined with Western medicine had no significant effects over Western medicine alone on anthropometric indices (BMI and WHR).

According to traditional Chinese medicine, the occurrence of PCOS is closely related to the kidneys, liver, spleen, and the Chong and conception channels, with kidney deficiency being the main cause followed by liver depression and spleen deficiency [47–49]. The combination of stasis blood, phlegm, and fluid retention, as well as water-dampness leads to a series of clinical symptoms. DKP is composed of the ingredients that have an effect on these symptoms, such as pilose antler (*Cornu Cervi Pantotrichum*), barbary wolfberry fruit (*Fructus Lycii*), and degelatined deer-horn (*Cornu Cervi Degelatinatum*), which warm the kidney, reinforce Yang, and nourish the liver and kidney. Chinese thorowax root (*Radix Bupleuri*), nutgrass galingale rhizome (*Rhizoma Cyperi*), Sichuan lovage rhizome (*Rhizoma Ligustici Chuanxiong*), and debarked peony root (*Radix Paeoniae Alba*) are combined to form the representative prescription Chaihu Shugan powder, which is used to disperse stagnated liver qi. Adding safflower (*Flos Carthami*), sanqi (*Radix Notoginseng*), motherwort fruit (*Fructus Leonuri*), and yanhusuo (*Rhizoma Corydalis*) promotes blood flow for regulating menstruation. Red ginseng (*Radix Ginseng Rubra*), largehead atractylodes rhizome (*Rhizoma Atractylodis Macrocephalae*), Indian bread (*Poria*), and licorice root (*Radix Glycyrrhizae*) are the famous prescriptions of Sijunzi decoction used for invigorating the spleen-stomach and replenishing qi, while debarked peony root (*Radix Paeoniae Alba*), prepared rehmannia root (*Radix Rehmanniae Preparata*), Chinese angelica (*Radix Angelicae Sinensis*), and Sichuan lovage rhizome (*Rhizoma Ligustici Chuanxiong*) are combined into a Siwu decoction as the basic prescription for nourishing blood for regulating menstruation, and the two prescriptions are combined to make the Bawu decoction, especially for benefiting qi and nourishing blood. Ass hide glue (*Colla Corii Asini*) is added to nourish Yin and tonify blood, and Baikal skullcap root (*Radix Scutellariae*) is added for clearing heat, removing dampness, and making the mixture tonic but not dry. Finally, the mixture is supplemented with honey (*Mel*) to reconcile the herbs. The whole prescription of DKP is rigorously formulated to harmonize Yin and Yang, coordinate Chong and the conception vessels, reinforce and eliminate in combination, nourish without stagnation and greasiness, and disperse without dispelling, all with the effect of nourishing the liver and the kidney, regulating menstruation and relieving Qi stagnation, and benefiting Qi and nourishing the blood.

Recent studies have also confirmed the efficacy of DKP in the treatment of PCOS. The chemical profiling of DKP by ultra high-performance liquid chromatography Q-exactive Orbitrap high-resolution mass spectrometry characterized over one hundred components and isomers, including amino acids, phenolic acids, lactones, terpenoids, alkaloids, saponins, flavonoids, and other compounds, among which paeoniflorin, ginsenosides, and notoginsenosides were present at high levels [18]. Modern pharmacological analysis suggests that paeoniflorin from debarked peony root (*Radix Paeoniae Alba*), rehmannia glutinosa polysaccharides from prepared rehmannia root (*Radix Rehmanniae Preparata*), and amino acids and proteins from ass hide glue (*Colla Corii Asini*) can enhance the hematopoietic function of the body [50–52]. Ginsenosides and notoginsenosides have been shown to be beneficial for insulin sensitivity and metabolic functions [18, 53]. Velvet antler polypeptides are one of the base components of the medicinal substances in pilose antler (*Cornu Cervi Pantotrichum*) and have fertility-enhancing effects [54]. The volatile oil components of largehead atractylodes rhizome (*Rhizoma Atractylodis Macrocephalae*) have both excitatory and inhibitory effects on the uterine smooth muscle to improve reproductive outcomes [55]. Ferulic acid in Chinese angelica (*Radix Angelicae Sinensis*) and Sichuan lovage rhizome (*Rhizoma Ligustici Chuanxiong*) and safflower yellow pigment in safflower (*Flos Carthami*) have shown inhibitory effects on platelet aggregation and release [56]. The lycium barbarum polysaccharide in the barbary wolfberry fruit (*Fructus Lycii*) can lower blood lipids and glucose levels [57]. Previous studies have shown that Chaihu Shugan powder, which is included in DKP, can regulate HPOA in women, thereby affecting serum hormone levels [58]. Sijunzi decoction has both hypoglycemic and hypolipidemic effects [59], and Siwu decoction has been proven to be effective in reversing infertility [60]. Thus, it can be seen that any of the Chinese herbal medicines or prescriptions contained in DKP exert their respective efficacies through their complex composition, which reflects the synergistic efficacy of Chinese herbal medicines among the composition of prescriptions.

Based on our analyses, DKP appears to have a positive effect on increasing the pregnancy rate, ovulation rate, and endometrial thickness in PCOS patients. Moreover, DKP also has effects on decreasing serum LH and T levels in patients with PCOS, and DKP may improve fertility through multiple possible mechanisms. PCOS is closely associated with HPOA functional disorders [2], including accelerated gonadotropin releasing-hormone pulsatile activity, increased secretion of pituitary LH, and increased ovarian secretion of T and estrogen, which can inhibit the development of follicles and oocytes, eventually contributing to ovulatory dysfunction [61]. A correlation exists between hyperandrogenemia and the development of IR and hyperinsulinemia [62], and excessive androgen also results in elevated levels of LH and FSH in PCOS patients [63]. DKP has a positive effect on restoring the feedback inhibition of HPOA, thus reducing the level of reproductive hormones, including T, LH, and FSH [16]. Furthermore, the implantation of fertilized eggs is impaired in PCOS patients because of changes in endometrial receptivity or to endometrial dysplasia because of inadequate exposure to progesterone [64]. HOXA10 is a characteristic marker of endometrial receptivity and is affected by the level of hormones [65]. An animal trial showed that DKP may play a role in improving endometrial receptivity by enhancing the expression of uterine HOXA10 [53]. In addition, ovarian fibrosis, which is characterized by the excessive proliferation of ovarian fibroblasts and deposition of extracellular matrix, is one of the pathophysiological causes of follicular dysplasia and ovulatory dysfunction in patients with PCOS [66]. DKP could be a promising approach to treating PCOS by downregulating the expression of transforming growth factor-beta 1 and connective tissue growth factor to interfere with extracellular matrix deposition [17].

IR occurs in 50% to 70% of women with PCOS [67], and the pathophysiology of type 2 diabetes mellitus is influenced by IR and abnormal glucose metabolism [68]. Most women with a family history of type 2 diabetes mellitus demonstrate impaired *β*-cell function or a subnormal disposition index [69]. In this meta-analysis, compared with COC alone, it was observed that DKP combined with COC significantly increased HOMA-*β* levels in PCOS patients, which suggested that DKP might have an effect on improving insulin sensitivity. We also assessed the effects of DKP on FBG and FINS in PCOS patients. However, we were unable to find any statistical difference between the intervention and control group. It is worth mentioning that Deng et al.‘s study [21] found that FBG was significantly decreased in PCOS patients after taking DKP for three months. Ginsenosides, one of the bioactive components of DKP, play a role in inhibiting the increase in blood glucose seen in PCOS patients with IR. The hypoglycemic effect of ginsenosides is mainly achieved by inhibiting hepatic gluconeogenesis, activating the AMPK signaling pathway, and stimulating glucose uptake [70]. Taken together, DKP may have a greater effect on insulin sensitivity than on IR, and DKP administration decreases glucose levels by increasing insulin sensitivity.

Clinical evidence shows a close association between cardiovascular disease and atherogenic dyslipidemia, which is characterized by elevated TC, TG, and LDL-C, and reduced HDL-C [71]. About 70% of women with PCOS in the U.S. suffer from dyslipidemia and possibly an increased risk of developing cardiovascular diseases [72, 73]. Notably, our observations provide a novel dimension to present evidence for the beneficial effects of DKP in mitigating dyslipidemia in women with PCOS. DKP significantly altered TC, TG, and FFA levels compared with the COC group in this meta-analysis. While there was inadequate evidence that DKP had a favorable influence on LDL-C and HDL-C in the present meta-analysis, some findings provided support for further investigation. One RCT reported a reduction in LDL-C and no increase in HDL-C in subjects with PCOS after the intake of DKP [22]. Overweight or obesity increases the risk for metabolic syndrome and cardiovascular disease in women with PCOS [74], and there is evidence that obesity may exacerbate IR, hyperandrogenism, or ovulatory dysfunction in PCOS, which can lead to infertility [75]. Although there was a lack of significant effect of DKP on BMI and WHR in our meta-analysis, some findings provided evidence for further exploration. The notoginsenosides found in DKP are potential active ingredients for the treatment of obesity by reducing lipid synthesis, inhibiting adipogenesis, increasing energy expenditure, and improving insulin sensitivity [76]. It can thus be seen that the results of this analysis were negative because of the insufficient survey of this critically important, yet largely ignored area, however, we cannot exclude a possible regulatory effect of DKP on anthropometric indices in PCOS patients.

Of the included trials, only two trials reported adverse reactions [43, 44]. One study [43] reported gastrointestinal or breast discomfort in the control group and the intervention group, however, the numbers of events were small and the symptoms were mild. In the other trial [44], there were three patients with abnormal serum biochemical indexes in the intervention group, however, there were ten patients presenting with abnormal indexes in the control group, and there were significant differences between the two groups. It should be mentioned that eight trials [10, 19, 21, 22, 27, 29, 34, 39, 40, 45] indicated that no adverse reactions occurred during the treatment. In addition, it is mentioned in the instructions of DKP that patients should stop taking it if they have a cold or flu. According to the current evidence, we believe that DKP is a relatively safe treatment. However, the long-term safety and efficacy of DKP in PCOS patients remains to be further explored.

### 4.2. Strengths and Limitations

This review has several strengths. We used metabolic indexes, lipid profiles, and anthropometric indexes as new evaluation indicators to discuss the efficacy of DKP on PCOS for the first time, consequently providing more possible therapies for PCOS. A previous meta-analysis [77], which only involved seven studies (with 658 participants), focused on the effectiveness of the combination of DKP and western medicine in ameliorating reproductive issues (ovulation rate, pregnancy rate, endometrial thickness, and hormone parameters), however, it did not perform an analysis of the effectiveness in regulating metabolic function in patients with PCOS. Moreover, our research has been registered on PROSPERO, and all procedures were faithfully executed accordingly, thus increasing the credibility of our results.

However, a few limitations exist in our study. Firstly, the follow-up time of these trials was inadequate, and the majority patients involved in the studies accepted about three months of treatment, and there was no evaluation of long-term outcomes. The live birth rate bears a great role in infertility clinical trials and is recognized as the major outcome [78]. Because of the lack of live birth rate, the trials included in this meta-analysis were insufficient to comprehensively address the role of DKP on reproductive health. Secondly, the heterogeneity between studies may stem from limitations in the methodological quality of the 22 RCTs included, such as the inappropriate use of blinding and differences in allocation concealment. Thirdly, although there were no language restrictions in the search, all trials were conducted in China, and 21 studies were published in domestic journals, which may lead to publication bias. It has been shown that publication bias in Chinese medical journals exists objectively because of the fact that negative results from clinical studies are not easily published and trials with small sample sizes are published [79]. The limitations mentioned above may affect the reliability of this meta-analysis, and thus the results in this review need to be interpreted with caution.

### 4.3. Implications for the Future

Clinical studies on interventions using DKP for PCOS are gradually increasing. In our meta-analysis, 18 RCTs were published between 2012 and 2020, followed by 4 RCTs published in 2021, indicating that DKP is a relatively new treatment for PCOS and that this intervention has great research value. Furthermore, we hope that modern clinical research on Chinese patent herbal medicines (such as DKP) can be spread to other countries to obtain more high-quality evidence for the use of such medicines in the clinic. At present, there is no single drug that is capable of treating infertility and metabolic complications associated with PCOS. As a multicomponent drug compound, DKP may have a role in the treatment of PCOS through multitarget synergistic actions. Therefore, future research on DKP for PCOS should focus on metabolic outcomes to identify more therapeutic methods for treating PCOS.

## 5. Conclusion

In summary, our results indicate that DKP has a promising application in modifying the reproductive and metabolic abnormalities in patients with PCOS and may be used as a primary choice in conventional or complementary therapies for PCOS. However, considering the inherent limitations and heterogeneity among the studies analyzed here, our results should be interpreted with caution. We expect more prospective large-scale and well-designed RCTs with longer intervention durations to further determine the clinical efficacy and safety of DKP in treating PCOS.

## Figures and Tables

**Table 1:** The characteristics of the included studies.

Study ID	Language	Study design	Age (years)	Sample size	Diagnostic criteria	Interventions	Duration	Outcomes	Adverse reaction
Du 2019 [26]	Chinese	RCT	29.7 ± 2.2	30	Rotterdam	DKP + OID	To pregnancy	Pregnancy rate, ovulation rate, endometrial thickness, LH, FSH, T	NR
			29.5 ± 2.1	30		OID			

Hu 2012 [27]	Chinese	RCT	NR	30	Rotterdam	DKP + OID	6 months	Pregnancy rate, LH, FSH, T	None
				30		OID			

Li 2018 [28]	Chinese	RCT	NR	40	Rotterdam	DKP + OID	To pregnancy	Endometrial thickness, T	NR
				40		OID			

Ma 2018 [29]	Chinese	RCT	26.2 ± 4.0	40	Rotterdam	DKP + OID	3 months	Pregnancy rate, endometrial thickness, LH, FSH, T	None
			25.4 ± 4.2	40		OID			

Qin 2021 [30]	Chinese	RCT	30.21 ± 3.81	49	Not clearly described	DKP + OID	3 months	Pregnancy rate, LH, FSH	NR
			29.71 ± 3.46	49		OID			

Ren 2020 [31]	Chinese	RCT	28.7 ± 1.3	36	Rotterdam	DKP + OID	3 months	Pregnancy rate, endometrial thickness, LH, FSH, T	NR
			28.5 ± 1.2	36		OID			

Wang 2019 [32]	Chinese	RCT	28.36 ± 7.92	55	Not clearly described	DKP + OID	To pregnancy	Pregnancy rate	NR
			28.25 ± 6.12	55		OID			

Wei 2012 [33]	Chinese	RCT	28.35 ± 1.25	30	Rotterdam	DKP + OID	3 months	Pregnancy rate, ovulation rate, endometrial thickness	NR
			29.25 ± 1.65	30		OID			

Wei 2018 [19]	Chinese	RCT	29.33 ± 0.96	50	Rotterdam	DKP + OID	1 month	Pregnancy rate, ovulation rate, endometrial thickness	None
			28.22 ± 0.76	50		OID			

Wei 2020 [34]	Chinese	RCT	26.12 ± 3.54	45	Rotterdam	DKP + OID	3 months	Pregnancy rate, endometrial thickness, LH, FSH, T	None
			27.35 ± 3.29	45		OID			

Yu 2020 [35]	Chinese	RCT	30.54 ± 2.34	46	Not clearly described	DKP + OID	To pregnancy	Pregnancy rate	NR
			30.25 ± 2.14	45		OID			

Yuan 2019 [36]	Chinese	RCT	31.12 ± 0.28	34	Not clearly described	DKP + OID	To pregnancy	Pregnancy rate	NR
			30.23 ± 0.62	34		OID			

Yu 2020 [37]	Chinese	RCT	NR	50	Obstetrics and gynecology	DKP + OID	To pregnancy	Pregnancy rate, ovulation rate	NR
				50		OID			

Yu 2021 [38]	Chinese	RCT	27.57 ± 2.25	55	Not clearly described	DKP + OID	To pregnancy	Endometrial thickness, LH, FSH, T	NR
			27.21 ± 2.36	55		OID			

Zhai 2019 [39]	Chinese	RCT	26.15 ± 3.18	44	Rotterdam	DKP + OID	6 months	Pregnancy rate, LH, FSH, T	None
			25.96 ± 3.33	44		OID			

Chu 2020 [40]	Chinese	RCT	29.27 ± 3.59	30	Chinese obstetrics and gynecology association	DKP + OID + DYD	1 month	Pregnancy rate, ovulation rate, endometrial thickness	None
			29.17 ± 3.51	30		OID + DYD			

Xiang 2020 [41]	Chinese	RCT	31.05 ± 3.37	105	Not clearly described	DKP + COC + MET	3 months	Pregnancy rate, ovulation rate, LH, FSH, T, FINS, HOMA-*β*	NR
			30.25 ± 3.42	105		COC + MET			

Zhong 2021 [42]	Chinese	RCT	28.69 ± 1.75	44	Guidelines for diagnosis and treatment of PCOS in China	DKP + COC + MET	3 months	Pregnancy rate, ovulation rate, LH, FSH, T, FINS, HOMA-*β*	NR
			28.54 ± 1.69	43		COC + MET			

Chen 2016 [43]	Chinese	RCT	30.3 ± 1.8	40	Rotterdam	DKP + COC	3 months	LH, FSH, T	Yes
			30.2 ± 1.7	40		COC			

Yu 2021 [44]	Chinese	RCT	24.47 ± 4.05	30	Rotterdam	DKP + COC	3 months	Endometrial thickness, LH, FSH, T	Yes
			23.83 ± 3.32	30		COC			

Deng 2020 [10, 21, 22]	English	RCT	27.5 ± 3.4	35	Rotterdam	DKP	3 months	BMI, WHR, FBG, FINS, TC, TG, HDL-c, LDL-C	None
			27.2 ± 3.5	36		COC			
			26.7 ± 6.4	39		DKP + COC			

Zhang 2019 [45]	Chinese	RCT	28.02 ± 3.21	40	Rotterdam	DKP	3 months	Pregnancy rate, ovulation rate, LH, FSH, T	None
			28.18 ± 3.10	40		COC			
			27.12 ± 3.30	40		DKP + COC			

DKP, Dingkun pill; OID, ovulation inducing drugs; COC, combined oral contraceptives; DYD, dydrogesterone; MET, metformin; LH, luteinizing hormone; FSH, follicle stimulating hormone; T, testosterone; BMI, body mass index; WHR, waist-to-hip ratio; FBG, fasting blood glucose; FINS, fasting Insulin; HOMA-*β*, homeostasis model assessment-*β*, TC, total cholesterol; TG, triacylglycerol; HDL-C, high-density lipoprotein cholesterol; LDL-C, low-density lipoprotein cholesterol; NR, not reported.

**Table 2:** Data and analyses of RCTs included in this systematic review and meta-analysis.

Outcome or subgroup	Participants	Mean difference	95% CI	*P* Value
FBG				
* DKP vs. COC*	71	0.10	[−0.09, 0.29]	0.31
* DKP* *+* *COC vs. COC*	75	0.10	[−0.15, 0.35]	0.43

BMI				
* DKP vs. COC*	71	0.70	[−1.81, 3.21]	0.59
* DKP* *+* *COC vs. COC*	75	0.60	[−1.92, 3.12]	0.64

WHR				
* DKP vs. COC*	71	0.00	[−0.03, 0.03]	1.00
* DKP* *+* *COC vs. COC*	75	–0.01	[−0.04, 0.02]	0.47

TC				
* DKP vs. COC*	71	–0.37	[−0.72, −0.02]	0.04
* DKP* *+* *COC vs. COC*	75	0.18	[−0.16, 0.52]	0.31

TG				
* DKP vs. COC*	71	–0.85	[−1.50, −0.20]	0.01
* DKP* *+* *COC vs. COC*	75	–0.19	[−1.02, 0.64]	0.65

LDL-C				
* DKP vs. COC*	71	0.09	[−0.23, 0.41]	0.58
* DKP* *+* *COC vs. COC*	75	0.21	[−0.11, 0.53]	0.20

HDL-C				
* DKP vs. COC*	71	–0.35	[−0.55, −0.15]	0.0008
* DKP* *+* *COC vs. COC*	75	–0.04	[−0.29, 0.21]	0.75

FFA				
* DKP vs. COC*	71	–130.00	[−217.56, −42.44]	0.004
* DKP* *+* *COC vs. COC*	75	–67.00	[−157.12, 23.12]	0.15

DKP, Dingkun pill; COC, combined oral contraceptives; FPG, fasting blood glucose; BMI, body mass index; WHR, waist-to-hip ratio; TC, total cholesterol; TG, triacylglycerol; HDL-C, high-density lipoprotein cholesterol; LDL-C, low-density lipoprotein cholesterol; FFA, free fatty acid.

**Table 3:** Quality of the evidence of selected primary outcomes according to the GRADE Working Group.

			Quality of assessment			Number of patients			Effect		Quality	Importance
Number of studies	Study design	Risk of bias	Inconsistency	Indirectness	Imprecision	Publication bias	Experimental	Control	Relative (95% CI)	Absolute (95% CI)		
Pregnancy rate: *DKP* *+* *OID vs. OID*												
14	RCT	Serious^a^	No serious inconsistency	No serious indirectness	No serious imprecision	Suspected^d^	325/574 (56.6%)	178/574 (31%)	RR 1.83 (1.6 to 2.09)	257 more per 1000 (from 186 more to 338 more	㊉㊉◯◯LOW	CRITICAL

Pregnancy rate: *DKP* *+* *COC vs. COC*												
3	RCT	Serious^a^	No serious inconsistency	No serious indirectness	Serious^c^	Undetected	126/189 (66.7%)	91/188 (48.4%)	RR 1.38 (1.16 to 1.64)	184 more per 1000 (from 77 more to 310 more	㊉㊉◯◯LOW	CRITICAL

Ovulation rate: *DKP* *+* *OID vs. OID*												
5	RCT	Serious^a^	Serious^b^	No serious indirectness	Serious^c^	Undetected	168/196 (85.7%)	126/196 (64.3%)	RR 1.38 (1.03 to 1.84)	244 more per 1000 (from 19 more to 540 more	㊉◯◯◯VERY LOW	CRITICAL

Ovulation rate: *DKP* *+* *COC vs. COC*												
3	RCT	Serious^a^	No serious inconsistency	No serious indirectness	Serious^c^	Undetected	165/189 (87.3%)	131/188 (69.7%)	RR 1.23 (1.11 to 1.37)	160 more per 1000 (from 77 more to 258 more	㊉㊉◯◯LOW	CRITICAL

Endometrial thickness: *DKP* *+* *OID vs. OID*												
9	RCT	Serious^a^	Serious^b^	No serious indirectness	No serious imprecision	Undetected	356	356	—	WMD 2.5 higher (1.91 to 3.09 higher)	㊉㊉◯◯LOW	CRITICAL

CI, confidence interval; RR, risk ratio; WMD, weighted mean difference; RCT, randomized controlled trial; DKP, Dingkun pill; OID, ovulation inducing drugs; COC, combined oral contraceptives. ^a^Randomization allocation and the blinding are unclear. ^b^I^2^ value was large. ^c^Number of patients included was less than 400. ^d^Funnel plot indicated a significant asymmetry.

## Data Availability

The data used to support the findings of this study are available from the corresponding author upon request.

## Supplementary Materials

Supplementary Materials Table S1. Search strategy. Figures S1–S6. Results of subgroup analyses.
