# Supplementary material for: The Efficacy and Safety of Dingkun Pill in Women with Polycystic Ovary Syndrome: A Systematic Review and Meta-Analysis of Randomized Controlled Trials
Source: Evid Based Complement Alternat Med. 2022 Aug 24;2022:8698755. doi: 10.1155/2022/8698755 (PMC9433272; doi:10.1155/2022/8698755)

Supplementary Materials

Table S1. Search strategy.

| PubMed | (("Polycystic Ovary Syndrome"[Mesh]) OR ((((((((((((((Ovary Syndrome, Polycystic[Title/Abstract]) OR (Syndrome, Polycystic Ovary[Title/Abstract])) OR (Stein-Leventhal Syndrome[Title/Abstract])) OR (Stein Leventhal Syndrome[Title/Abstract])) OR (Syndrome, Stein-Leventhal[Title/Abstract])) OR (Sclerocystic Ovarian Degeneration[Title/Abstract])) OR (Ovarian Degeneration, Sclerocystic[Title/Abstract])) OR (Sclerocystic Ovary Syndrome[Title/Abstract])) OR (Polycystic Ovarian Syndrome[Title/Abstract])) OR (Ovarian Syndrome, Polycystic[Title/Abstract])) OR (Polycystic Ovary Syndrome 1[Title/Abstract])) OR (Sclerocystic Ovaries[Title/Abstract])) OR (Ovary, Sclerocystic[Title/Abstract])) OR (Sclerocystic Ovary[Title/Abstract]))) AND (((dingkun dan[Title/Abstract]) OR (dingkun pill[Title/Abstract])) OR (dingkun[Title/Abstract])) |
| --- | --- |
| EMBASE | ('ovary polycystic disease'/exp OR 'polycystic ovary syndrome':ti,ab,kw OR 'Ovary Syndrome, Polycystic':ti,ab,kw OR 'Syndrome, Polycystic Ovary':ti,ab,kw OR 'Stein-Leventhal Syndrome':ti,ab,kw OR 'Stein Leventhal Syndrome':ti,ab,kw OR 'Syndrome, Stein-Leventhal':ti,ab,kw OR 'Sclerocystic Ovarian Degeneration':ti,ab,kw OR 'Ovarian Degeneration, Sclerocystic':ti,ab,kw OR 'Sclerocystic Ovary Syndrome':ti,ab,kw OR 'Polycystic Ovarian Syndrome':ti,ab,kw OR 'Ovarian Syndrome, Polycystic':ti,ab,kw OR 'Polycystic Ovary Syndrome 1':ti,ab,kw OR 'Sclerocystic Ovaries':ti,ab,kw OR 'Ovary, Sclerocystic':ti,ab,kw OR 'Sclerocystic Ovary':ti,ab,kw OR 'PCOS':ti,ab,kw) AND ('dingkun dan':ti,ab,kw OR 'dingkun pill':ti,ab,kw OR 'dingkun':ti,ab,kw) |
| Cochrane library | #1 MeSH descriptor: [Polycystic Ovary Syndrome] explode all trees  #2 (Polycystic Ovary Syndrome):ti,ab,kw  #3 Ovary Syndrome, Polycystic):ti,ab,kw  #4 (Syndrome, Polycystic Ovary):ti,ab,kw  #5 (Stein-Leventhal Syndrome):ti,ab,kw  #6 (Stein Leventhal Syndrome):ti,ab,kw  #7 (Syndrome, Stein-Leventhal):ti,ab,kw  #8 (Sclerocystic Ovarian Degeneration):ti,ab,kw  #9 (Ovarian Degeneration, Sclerocystic):ti,ab,kw  #10 (Sclerocystic Ovary Syndrome):ti,ab,kw  #11 (Polycystic Ovarian Syndrome):ti,ab,kw  #12 (Ovarian Syndrome, Polycystic):ti,ab,kw  #13 (Polycystic Ovary Syndrome 1):ti,ab,kw  #14 (Sclerocystic Ovaries):ti,ab,kw  #15 (Ovary, Sclerocystic):ti,ab,kw  #16 (Sclerocystic Ovary):ti,ab,kw  #17 (PCOS):ti,ab,kw  #18 #1 OR #2 OR #3 OR #4 OR #5 OR #6 OR #7 OR #8 OR #9 OR #10 OR #11 OR #12 OR #13 OR #14 OR #15 OR #16 OR #17  #19 (dingkun dan):ti,ab,kw  #20 (dingkun pill):ti,ab,kw  #21 (dingkun):ti,ab,kw  #22 #19 OR #20 OR #21  #23 #18 AND #22 |
| Web of Science | TS=("polycystic ovary syndrome" or "Ovary Syndrome, Polycystic" or "Syndrome, Polycystic Ovary" or "Stein-Leventhal Syndrome" or "Stein Leventhal Syndrome" or "Syndrome, Stein-Leventhal" or "Sclerocystic Ovarian Degeneration" or "Ovarian Degeneration, Sclerocystic" or "Sclerocystic Ovary Syndrome" or "Polycystic Ovarian Syndrome" or "Ovarian Syndrome, Polycystic" or "Polycystic Ovary Syndrome 1" or "Sclerocystic Ovaries" or "Ovary, Sclerocystic" or "Sclerocystic Ovary" or PCOS) AND TS=("dingkun dan" or "dingkun pill" or "dingkun") |
| CNKI | SU=(定坤丹+定坤) AND SU=(多囊卵巢综合征+polycystic ovarian syndrome+polycystic ovary syndrome+women with polycystic ovary syndrome+多囊性卵巢综合症+多囊性卵巢综合征+多囊卵巢综合证+多囊卵巢综合症+多囊性卵巢+PCOS)  ***English translation:***  SU=(dingkun dan + dingkun) AND (polycystic ovarian syndrome + polycystic ovary syndrome + women with polycystic ovary syndrome + polycystic ovary + PCOS) |
| Wanfang | 主题:(多囊卵巢综合征 or polycystic ovarian syndrome or polycystic ovary syndrome or women with polycystic ovary syndrome or 多囊性卵巢综合症 or 多囊性卵巢综合征 or 多囊卵巢综合证 or 多囊卵巢综合症 or 多囊性卵巢) and 主题:(定坤丹 or 定坤)  ***English translation:***  Subject:(polycystic ovarian syndrome or polycystic ovary syndrome or women with polycystic ovary syndrome or PCOS) and ("dingkun dan" or "dingkun") |
| VIP | M=(多囊卵巢综合征 OR polycystic ovarian syndrome OR polycystic ovary syndrome OR women with polycystic ovary syndrome OR 多囊性卵巢综合症 OR 多囊性卵巢综合征 OR 多囊卵巢综合证 OR 多囊卵巢综合症 OR 多囊性卵巢) AND M=(定坤丹 OR 定坤)  ***English translation:***  (M=(polycystic ovarian syndrome OR polycystic ovary syndrome or women with polycystic ovary syndrome OR PCOS) AND M=(dingkun dan or dingkun) |
| Chinese Biomedical Literature database | ("定坤丹"[不加权:扩展] OR 定坤丹 OR 定坤) AND ("多囊卵巢综合征"[不加权:扩展] OR 多囊卵巢综合征 OR polycystic ovarian syndrome OR polycystic ovary syndrome OR women with polycystic ovary syndrome OR 多囊性卵巢综合症 OR 多囊性卵巢综合征 OR 多囊卵巢综合证 OR 多囊卵巢综合症 OR 多囊性卵巢 OR PCOS)  ***English translation:***  ("Dingkundan" [unweighted: extended] OR Dingkun OR Dingkun) AND ("Polycystic ovary syndrome" [unweighted: extended] OR polycystic ovary syndrome OR polycystic ovarian syndrome OR polycystic ovary syndrome OR women with polycystic ovary syndrome OR polycystic ovary OR PCOS) |

Figure S1. Subgroup analysis of the effects of DKP duration on pregnancy rate


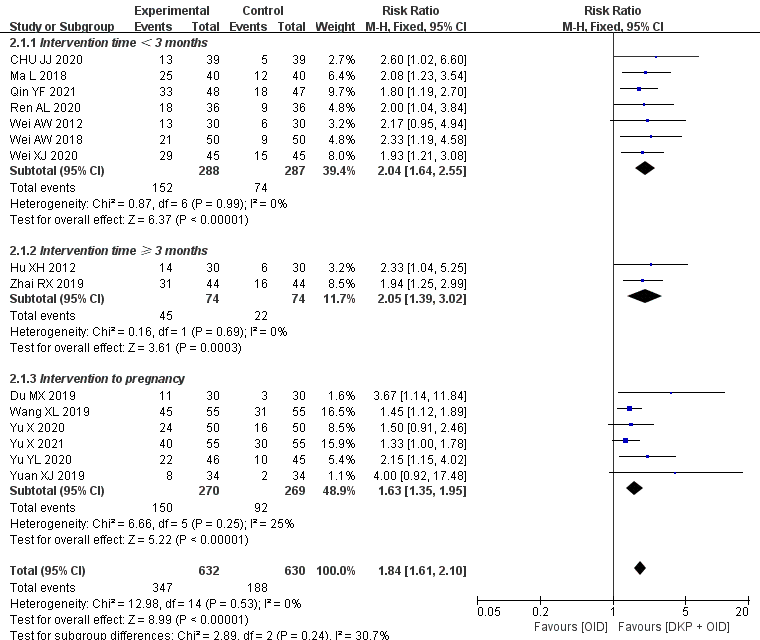


Figure S2. Subgroup analysis of the effects of DKP duration on ovulation rate


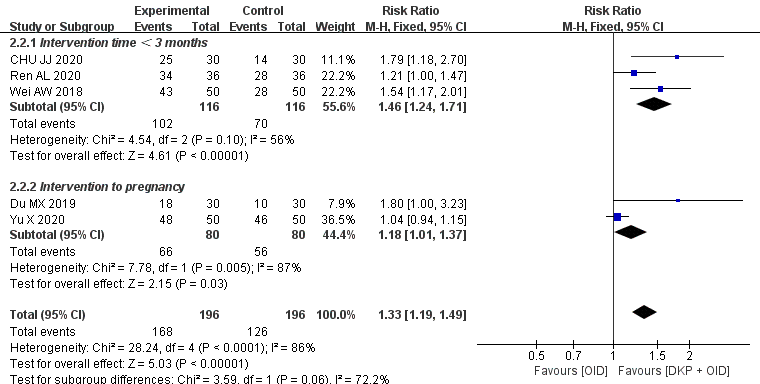


Figure S3. Subgroup analysis of the effects of DKP duration on endometrial thickness


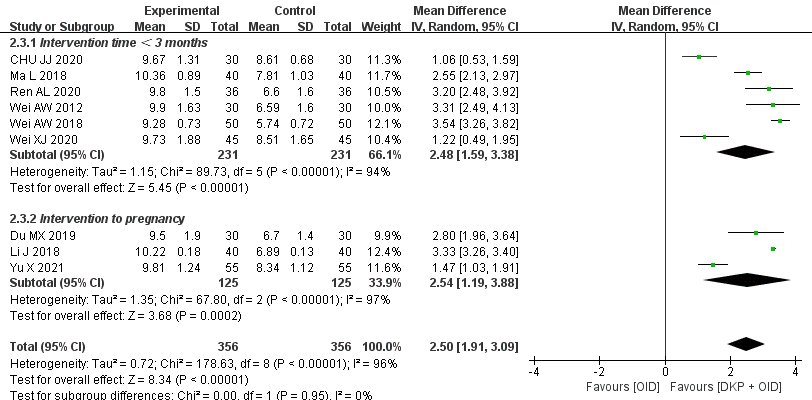


Figure S4. Subgroup analysis of the effects of DKP duration on LH


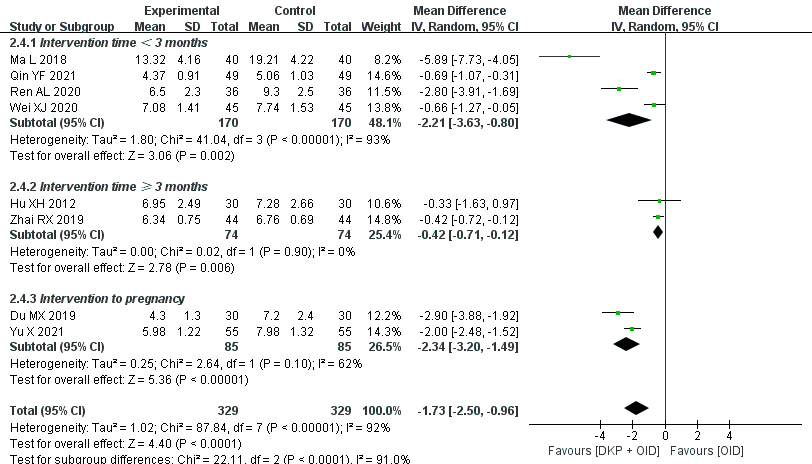


Figure S5. Subgroup analysis of the effects of DKP duration on FSH


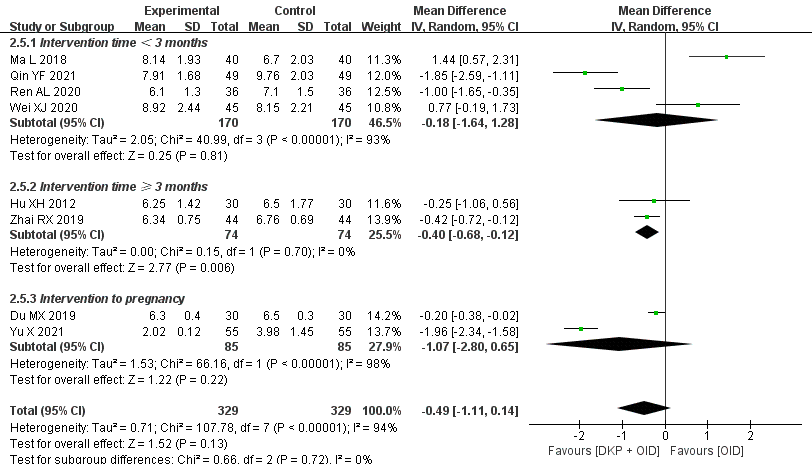


Figure S6. Subgroup analysis of the effects of DKP duration on T


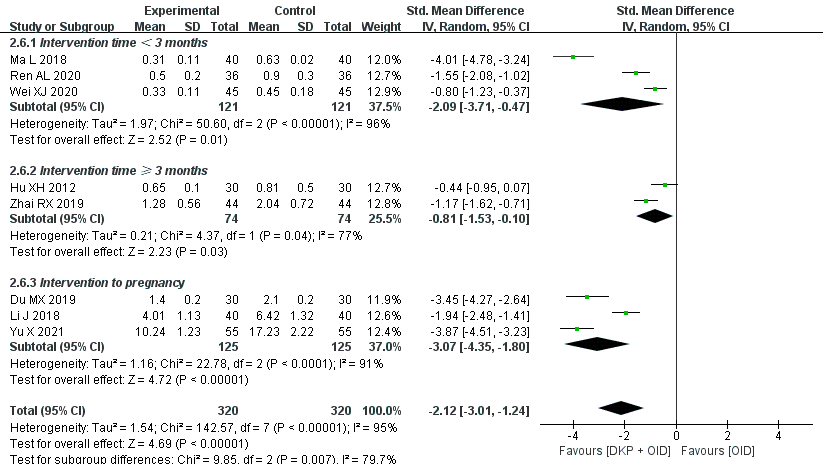

Supplement: Supplementary Materials — Table S1. Search strategy. Figures S1–S6. Results of subgroup analyses. [file 8698755.f1.docx]
